# Gut microbiota, probiotics, and migraine: a clinical review and meta-analysis

**DOI:** 10.22514/jofph.2025.043

**Published:** 2025-09-12

**Authors:** Olga Grodzka, Izabela Domitrz

**Affiliations:** ^1^Department of Neurology, Faculty of Medicine and Dentistry, Medical University of Warsaw, 01-809 Warsaw, Poland; ^2^Doctoral School, Medical University of Warsaw, 02-097 Warsaw, Poland

**Keywords:** Gut microbiota, Intestinal microbiota, Microbiome, Migraine, Probiotics

## Abstract

Migraine is a primary headache disorder affecting about 14% of the global population. The knowledge about migraine pathophysiology is increasing constantly; however, there are still many unknowns and uncertainties. Intestinal microbiota builds the gut environment together with metabolites and the immune system. Its connections with disorders outside the digestive system have been described, mainly neuropsychiatric diseases, due to the existence of the microbiota-gut-brain axis. Therefore, it is suggested that migraine is also correlated with changes in the microbiome. The review aimed to summarize the available literature related to the topic. We performed an electronic article search through the Embase Database and PubMed Database, and included 14 articles after analysis under the Preferred Reporting Items for Systematic Reviews and Meta-analyses (PRISMA) 2020 guidelines. Subsequently, a meta-analysis of randomized controlled clinical trials summarizing probiotics’ effect on migraine prevention was conducted based on the same guidelines and resulted in including 2 adequate trials. Microbiome alterations have been observed in migraine patients with an influence on clinical presentation. Preclinical studies suggested a direct connection between migraine and microbiome changes. The meta-analysis has shown the influence of probiotics on migraine frequency (*p* = 0.003; Hedges’ *g* = 1.22; standard error (SE) = 0.41), and no impact on migraine severity (*p* = 0.069; Hedges’ *g* = 1.10; SE = 0.61) and attacks’ duration (*p* = 0.149; Hedges’ *g* = 0.18; SE = 0.15). However, the former was close to the statistical significance. The following work demonstrates a correlation between migraine and microbiome, which has a putative positive impact on migraine management. Moreover, probiotic supplementation can alleviate migraine symptoms. However, the main limitation is the limited number of studies, together with high heterogeneity and limited methodological consistency in the meta-analysis.

## 1. Introduction

Migraine is a common disorder with a prevalence established at 14% of the population worldwide [1]. According to The International Headache Classification, 3rd edition [2], migraine is usually divided into three main types: migraine with aura, migraine without aura, and chronic migraine. Noteworthy, migraine remains one of the main causes of disability globally [3], leading among women aged 15–49 [4]. The migraine pathophysiology is linked with several mechanisms, including lack of habituation and central sensitization [5], the role of the trigeminal-cervical complex, brain areas, such as the thalamus, hypothalamus, and cortex, and peptides: calcitonin gene-related peptide (CGRP), pituitary adenylate cyclase-activating polypeptide (PACAP) [6], and vasoactive intestinal peptide (VIP) [7]. The crucial point of the abovementioned elements, especially in the view of this work, is central sensitization, which arises when the excitability of neurons increases in central nociceptive pathways, being a prolonged but fully reversible process. This phenomenon can be caused by frequent attacks and medication overuse, not only in chronic migraine, but also in any chronic pain disease [5, 8]. One of the factors contributing to central sensitization in chronic pain is the intestinal microbiome, including metabolites, neuromodulators, neuropeptides, and neurotransmitters derived from the bacteria [9, 10]. The understanding of its molecular bases is constantly advancing. Although research on migraine pathogenesis and molecular bases continues, the knowledge is still limited, and hence, not all diagnostic and therapeutic options have been discovered. Further research may lead to future achievements and facilitate migraine management and understanding.

The intestinal microbiota has recently become an area of great interest. Its balance with diverse metabolites and the human immune system builds a whole gut environment. Any alterations may appear either as a result of or cause of health problems [11]. Moreover, beneficial changes caused by probiotic intake are likely to maintain or even improve one’s health [12]. Naturally, the role of gut microbiota has been comprehensively studied and shown in gastrointestinal diseases [13, 14]; however, it goes further outside the digestive system. Several studies have focused on the value of the microbiome in psychiatric [15, 16] or neurological diseases [17, 18]. Those findings are closely connected to the microbiota-gut-brain axis.

Connections between the gut and brain have been found crucial to maintaining homeostasis by regulating both the central nervous system and the enteric nervous system [19]. Among the diverse endocrine, immune, and neuronal signals between the intestines and the brain stand the vagal nerve, intestinal hormones, and cytokines [20]. Moreover, signals can be derived in opposite directions: afferent (“gut-brain”) and efferent (“brain-gut”). The responses are towards external factors, such as food and environmental antigens, and internal factors, such as microbiota with its molecular products [21] (Summarized in Fig. 1).

**Fig. 1.** A graphical presentation of the main elements contributing to the gut-brain axis functioning.

Multiple studies have examined the connection between the digestive and neurological systems. Altered microbiota in diverse diseases potentially has value in their management, including diagnosis, treatment, and prevention [22]. Furthermore, a perspective on the microbiota-gut-brain axis may help better understand some pathogenetic aspects [23, 24].

Finally, some recent studies have focused on the role of the microbiome in migraine management and pathogenesis, seeking possible correlations [25, 26]. Also, another issue that should be raised is the role of probiotics in migraine management; however, research on this specific topic remains scarce. Probiotics taken regularly are mainly suggested to be beneficial in migraine prophylaxis, affecting the course of the disease; nevertheless, their role is much more complex [27, 28]. Thus, this work summarizes the current knowledge from available literature on this topic and provides additional analysis of probiotics’ use in migraine prevention. The aim of the review was to analyze gut microbiome alterations and probiotics influence (I as intervention in PICO framework) on the migraine course (O as outcome) in migraine patient population (P as population) in comparison to healthy controls (C as comparison). We hypothesize that, firstly, the changes observed in intestinal microbiota are inseparable from migraine incidence and course, and, secondly, probiotics may be used as supportive agents in the preventive treatment of migraine.

## 2. Materials and methods

In the process of preparing, this study was divided into two major parts: (i) a review analyzing microbiome changes in migraine as a result of the original idea, and secondary, (ii) a meta-analysis of the effects of probiotics on migraine course to exhaust the topic. The review was performed narratively; however, in a more systematic manner, following the Preferred Reported Items for Systematic Reviews and Meta-analyses (PRISMA) 2020 guidelines [29] to avoid the risk of bias. Two Databases, the PubMed Database and Embase Database, were searched by the first author in each part of the work (Fig. 1) and supervised by the senior author. The review was registered in PROSPERO (ID CRD42024532026); however, it should not be considered strictly a systematic review, since, among other, only one author performed the initial screening of the potential studies.

The methodological quality of the articles was assessed with the methodological quality appraisal tool [30], which included the criteria for all crucial aspects of research methodology: outcome measures, background or literature review, sample description, study design and methodology, and conclusions. Each criterion was evaluated for 0 or 1 points. Low-quality studies with a score of 2 or fewer points were not considered; however, no such situation happened. The quality assessment of the included studies was performed by the first author (OG) with additional confirmation by the second author. There were no discrepancies between the authors.

### 2.1 A review

The search query for the review was as follows: (migraine) AND (intestinal microbiota OR gut microbiota OR intestinal microbiome OR gut microbiome).

#### 2.1.1 Inclusion and exclusion criteria

Only primary research, precisely, randomized controlled trials, cohort studies, case-control studies, Mendelian randomized analysis studies, and basic preclinical research were included. Therefore, narrative and systematic reviews, meta-analyses, case reports, case series, commentaries, letters to the editors, and editorials were not included. However, conference abstracts were not taken into consideration.

The Population, Intervention, Comparison, and Outcome (PICO) characteristics [31] for eligibility were applied. Therefore, the included publications should have focused on migraine patients in general or divided them into subgroups (Population) compared to healthy individuals or patients without migraine (Comparison). Moreover, an analysis of the participants’ microbiome should have been performed (Intervention), and the correlation with migraine assessed (Outcome). Therefore, studies focusing only on headaches other than migraines were not considered. Consistently, research without mentioning the intestinal microbiome at any point of the study did not meet the inclusion criteria. Furthermore, studies that remained contrary to the International Classification of Headache Disorders guidelines were not included. Articles written in languages other than English were excluded.

#### 2.1.2 Selection process

The selection process of the studies appropriate for the review was performed by the first author (OG). Additionally, all the studies were analyzed and approved by the second author (ID), with a constant possibility to suggest additional research. Discrepancies, if any, were resolved through discussion between the authors. An initial search led to the indentification of 244 studies, from which 61 were removed as duplicates. Therefore, 183 records were left with to screen. In addition, 136 articles were excluded by title or type, another 29 by abstract, and later, 4 were excluded after the full-text analysis. As a result, 14 studies were left and have been included in this review (Fig. 2a).

#### 2.1.3 Synthesis of the results

Data analysis of the studies was performed after the selection process with quality assessment. A narrative format of the extracted data was applied for the review.

### 2.2 Meta-analysis

A thorough analysis of the review articles, both included and not included, led us to additional insights into the topic. Consequently, in the second part of this work, our interests focused on the influence of probiotics on migraine courses. We decided to conduct an additional search of the PubMed and Embase databases using another query: “migraine” AND “probiotics”, which was used to conduct a systematic review and subsequent meta-analysis. The full search query, search dates, and language limits for both the systematic review and meta-analysis have been presented in **Supplementary Table 1**.

#### 2.2.1 Inclusion and exclusion criteria

The PICO characteristics were applied anew. The analyzed population was similar to the previous part, including migraine sufferers, but with probiotics supplementation. A comparison must have been performed with participants with migraine receiving placebo therapy instead. As described, the probiotics or placebo intervention was applied, and after an adequate time, the outcomes were precisely assessed, including the influence on migraine characteristics, considered as primary endpoints: pain severity, frequency, and attack duration. All outcomes were measured with mean difference and standard deviation. Only randomized controlled clinical trials were included. Moreover, the included studies analyzed only data from adult patients, both males and females, to maintain consistency in further statistical analysis. Additionally, all chosen research had to present sufficient data and be written in English. Therefore, studies other than randomized controlled trials conducted on pediatric populations with missing data or written in a language other than English were excluded.

Grey literature was not systematically searched, as the review aimed to synthesize evidence from peer-reviewed randomized controlled trials to ensure methodological rigor and data quality. While this may limit comprehensiveness, it reduces the potential inclusion of non-standardized or non-validated data sources that could bias the meta-analytic estimates.

#### 2.2.2 Selection process

197 studies were found in the initial search of the two databases; however, 35 were duplicates. After screening of the remaining articles, 140 were removed by title or type, 15 based on abstract, and 5 after analysis of the full text. Among those 5 excluded articles, two included a pediatric population, one did not have any comparison or control group, one did not mention any of the analyzed migraine features, and the other one did not contain complete data to conduct adequate statistical analysis. Finally, after reliable and thorough screening following the established criteria, 2 studies were analyzed (Fig. 2b). The Grading of Recommendations Assessment, Development and Evaluation (GRADE) summary of findings, including the assessment of the certainty of evidence, is presented in **Supplementary Table 2**.

**Fig. 2.** A flowchart of the selection process according to the PRISMA guidelines representing the selection process for the first (2a) and second (2b) parts of the study. n, number of studies.

#### 2.2.3 Statistical analysis

Statistical analysis was performed in Microsoft Excel 2016 (Microsoft Corporation, Redmond, WA, USA) using the workbooks and the user manual licensed under the Creative Commons Attribution-NonCommercial-ShareAlike 4.0 International License [32]. The Excel workbooks provided all the necessary calculations, including heterogeneity analysis, random or fixed effect meta-analysis models, Hedges effect size measures, and 95% confidence intervals. The measures analyzed in the studies were the mean difference and standard deviation. The results were also illustrated by forest plot diagrams. A *p*-value of < 0.05 was considered for statistical significance.

## 3. Results

The correlation between migraine and intestinal microbiome was analyzed in several different ways, including different types of studies (clinical and preclinical), various sources of data (patients recruited directly or from genome-wide association studies (GWAS) databases), and focusing on the microbiome in general or on particular species and genera. To analyze all included studies thoroughly and not miss any critical conclusions, four main issues of the review have been highlighted in separate paragraphs.

As mentioned before, literature analysis also drew our attention to the matter of probiotics use in migraine prophylaxis, raised in some of the studies not included in the primary review. Although microbiome alterations were not mentioned in those studies, we found them interesting and closely related to the topic, as probiotics are undoubtedly known to influence intestinal microbiomes. Microbiome and probiotics issues are firmly connected, overlapping each other; thus, analyzing them collectively allows a comprehensive view of the subject. Therefore, we have decided to perform a meta-analysis summarizing the data about the role of probiotics in migraine. Another search was conducted and precisely described in the paragraph: “Additional findings after the literature review: probiotics in migraine prophylaxis”.

### 3.1 How does migraine correlate with microbiome diversity?

The most frequently raised issue in the topic of migraine and microbiome was the relationship between migraine and microbiome α- and β-diversity. α-diversity stands for microbiota richness and evenness, and β-diversity represents the changes in species diversity between different environments. Additionally, the third type of diversity, γ-diversity, describes the total observed richness [33]. The connection between migraine and microbiome diversity was found in the studies described in the following paragraph; nevertheless, the uncertainty regards the specific mechanism underlying it. The research did not show the direction of the possible impact; either the migraine generates the microbiome alterations, or the opposite. Furthermore, both interactions can co-exist. Studies exploring the pathogenesis of the correlation should, thus, be conducted.

Two research groups analyzed pediatric populations with migraine regarding microbiome alterations and diversity. First, Bai *et al*. [34] used data from the American Gut Project to investigate the association between migraine and microbiota in a pediatric population. Both α- and β-diversity showed lower diversity in the microbiota of migraine patients in comparison to healthy controls (*p* < 0.01 in Shannon Index and Faith phylogenetic diversity for α-diversity assessment; *p* = 0.001 in weighted UniFrac distance and Bray-Curtis dissimilarity index for β-diversity evaluation). Moreover, several phyla levels increased or decreased in children with migraines compared to those without. Furthermore, among the bacteria with significantly increased abundance in migraine sufferers were those involved in the inflammatory response, such as *Eggerthella*, *Sutterella* and *Eubacterium*. The second team, Liu *et al*. [35], conducted a study on the pediatric population, also comparing individuals with migraines with healthy controls matched by sex and age. The study contained two main parts; however, only one focused on the alterations in gut microbiota presented by migraine patients. Gut dysbiosis, understood as a decrease in microbiota diversity, was observed in children with migraine. It was also shown that among the analyzed bacteria, nine genera demonstrated significantly higher abundance, while 16 genera had lower abundance in migraine patients compared to the control group. Moreover, considering sex differences, eight genera differ only in the female population and seven genera in the male population. The second part of the study focused on the putative role of tryptophan metabolism in pediatric migraine. Although this topic isn’t directly related to our review, it is worth noting that such a relation has been observed.

From our review, two other studies focused on adult populations. Chen *et al*. [36] investigated elderly women with migraine and compared them to healthy individuals. Both groups were shown to differ significantly in α-diversity but not in microbiome richness. Moreover, 21 and 22 species were enriched in migraine sufferers and healthy controls, respectively. Interestingly, from the functional point of view, the migraine group presented increased gut-brain modules, including kynurenine degradation and γ-aminobutyric acid (GABA) synthesis, in comparison to the control group. Subsequently, in their study, Geisler *et al*. [37] compared microbiome diversity between neurological patients and healthy individuals. Among those with neurological diseases, over 50% of cases were migraine. Moreover, 135 out of 148 patients suffering from chronic pain were diagnosed with migraine. The abundance of 51 operational taxonomic units (OTUs) significantly differed among neurological patients compared to healthy controls. The main phylum appeared to be *Firmicutes* (40 OTUs), followed by *Bacteroidetes* (6 OTUs) and *Proteobacteria* (4 OTUs). However, this observation requires further studies since after adjustment for the number of OTUs and other possible affecting factors (such as lifestyle, diet, medications and vitamins, body mass index, age and sex), there were no longer significant differences. Furthermore, regardless of whether the adjustment was performed, neurological subgroups did not differ in abundance. Another interesting point of the study was the differences in both α- and β-diversity between neurological patients and healthy controls, and what is more, between specifically chronic pain patients and healthy controls. Finally, similar differences were also observed between neurological subgroups. Unfortunately, the comprehensive data stratified specifically for migraine patients is lacking.

Finally, one study found no differences in diversity between migraine sufferers and healthy individuals. Yong *et al*. [38] carried out a study in which researchers not only compared migraine patients to healthy controls but also included the migraine classification into two types: episodic and chronic migraine. It was demonstrated that the abundance of several bacteria differed between all the three compared groups. Surprisingly, there were no significant differences in microbiota α- and β-diversity. Another important issue covered by the researchers was correlations between microbiota genera and the frequency or severity of headaches in migraine patients. It was shown that a higher abundance of *PAC000195-g* genera was correlated with lower headache frequency, while a higher abundance of *Agathobacter*—with decreased headache severity. The influence on clinical presentation will be described more thoroughly in the next paragraph. All studies mentioned in this paragraph, with additional information, were summarized in Table 1 (Ref. [34, 35, 36, 37, 38]).

**Table 1.** A summary of studies focusing on alterations of microbiome in migraine in general.

Ref.	Year	Population	Comparison	Results	Methods
[34]	2023	40 pediatric migraine patients 15,279 patients (data for intestinal microbiota)	341 healthy controls	↓ Microbiome diversity in migraine patients than in healthy controls ↑ Abundance of inflammation-related bacteria in migraine patients than in healthy controls	Data from American Gut Project Microbiome α-diversity with Shannon Index, Pielou Index, Faith phylogenetic diversity Microbiome β-diversity by weighted UniFrac distance, Bray-Curtis dissimilarity index, and principal coordinates analysis
[35]	2023	33 migraine pediatric patients	42 healthy controls	↑ Of 9 genera and ↓ of 16 genera in migraine patients compared to healthy controls Intestinal dysbiosis in migraine patients compared to healthy controls	Based on GMrepo database Microbiome α-diversity with Shannon Index and Simpson Index Microbiome overall diversity by principal coordinates analysis Bacterial abundance with Wilcoxon rank-sum test
[36]	2020	54 migraine female patients	54 healthy controls	↓ Microbiome α-diversity in migraine patients compared to healthy controls at genus and species levels No statistical difference in species richness between both groups 21 enriched species in study group 22 enriched species in healthy controls	Microbiome by shotgun sequencing and mapping with SOAP v2.22 Metaphlan2 to obtain taxonomic profiles Microbiome α-diversity with Shannon Index MWAS to find correlations between migraine and microbiome
[37]	2021	238 neurological patients: 135 migraine patients (among 148 chronic pain patients) 103 non-migraine patients	612 healthy controls	Significantly different abundance of 51 OTUs in study group compared to healthy controls No statistical difference in OTUs between neurological subgroups ↓ Microbiome α-diversity, richness and ↑ evenness in neurological patients than in healthy controls ↓ Microbiome α-diversity, richness and ↑ evenness in chronic pain patients than in healthy controls Significant difference in β-diversity between study group and healthy controls and between neurological subgroups	Microbiome by 16s rRNA sequencing Microbiome α-diversity with Shannon Index Microbiome β-diversity by principal coordinates analysis and Bray-Curtis dissimilarity index
[38]	2023	42 episodic migraine patients 45 chronic migraine patients	43 healthy controls	No statistical difference in microbiome diversity between study groups and healthy controls ↑ Of 1 genera and ↓ of 10 genera in episodic migraine patients than in healthy controls ↑ Of 4 genera and ↓ of 14 genera in episodic migraine patients than in healthy controls ↑ Of 1 genera in chronic migraine patients than in episodic migraine patients ↑ Of 5 genera in episodic migraine patients than in chronic migraine patients	Microbiome by PCR and partial, targeted sequencing of 16S rRNA V3–V4 region microbiome α-diversity with Shannon Index, Simpson Index, Chao1 Index Microbiome β-diversity by principal coordinates analysis (with weighted UniFrac distance), unweighted UniFrac distance and Bray-Curtis dissimilarity index

*↑, increase; ↓, decrease; MWAS, metagenome-wide association studies; OTUs, operational taxonomic units; PCR, polymerase chain reaction; Ref., reference; RNA, ribonucleic acid; rRNA, ribosomal RNA; UniFrac, unique fraction metric.*

### 3.2 Correlations between gut microbiome and clinical presentation

In several studies, correlations between intestinal microbiome changes and clinical presentation of patients were included. Kopchak *et al*. [39] investigated one hundred participants divided into two groups: (i) patients with diagnosed migraine and (ii) healthy individuals. Interestingly, apart from several bacteria found to be altered in migraine patients in comparison to healthy controls, also some fungi and one virus—Herpes simplex were observed to differ between the study and control groups. Moreover, correlations were observed between particular microbiota and clinical features such as pain severity (measured by visual-analogue scale (VAS); *p* = 0.046 for *Alcaligenes* spp.; *p* = 0.049 for *Eggerthella*), frequency of attacks (*p* = 0.046 for *Alcaligenes* spp.), impact of headaches on patient’s life (assessed in Migraine Disability Assessment (MIDAS) questionnaire; *p* = 0.035 for *Clostridium coccoides*), and psycho-emotional status (evaluated in Beck Depression Inventory (BDI); *p* = 0.007 for *Alcaligenes* spp.; *p* = 0.025 for *Clostridium coccoides*).

In another research, the issue of correlation between migraine and intestinal disorders was raised; therefore, a comparison was conducted for patients with irritable bowel syndrome (IBS) and non-healthy controls, as in the previous studies. Noteworthy, there is a correlation between IBS and migraine, which results in their more frequent co-occurrence. Liu *et al*. [40] intended to study the microbiota differences in migraine patients suffering from IBS. The comparison between participants with migraine and IBS and those with only IBS revealed changes in microbiota structure but not richness and diversity in the study group compared to the control group. Furthermore, the clinical characteristics showed no differences between the groups. Although five genera presented with altered abundance in migraine patients compared to healthy controls, only two genera remained significant after linear discriminant analysis (LDA). More precisely, higher and lower abundance of genus *Parabacteroides* and *Paraprevotella*, respectively, were observed in patients affected by migraine and IBS in comparison to those with IBS only.

A slightly different study design was prepared by Georgescu *et al*. [41], who studied female patients suffering from migraine with aura. The group was divided into two subgroups: (i) patients with gut dysbiosis and (ii) patients without gut dysbiosis. Subsequently, both subgroups were compared to each other, showing increased migraine severity and duration in the dysbiosis-positive group. Moreover, the dysbiosis group presented significantly higher carotid intima-media thickness (CIMT). Noteworthy, CIMT is known as a marker of atherosclerosis, which was proven to be related to migraine. Finally, the dysbiosis group presented with increased tumor necrosis factor-alpha (TNF-α) levels, a proinflammatory cytokine. All studies mentioned in this paragraph, with additional information, were summarized in Table 2 (Ref. [39, 40, 41]).

**Table 2.** A summary of research describing correlations of microbiota with migraine clinical presentation.

Ref.	Year	Population	Comparison	Results	Methods
[39]	2022	Migraine patients	Healthy controls	Alterations in abundance of species: *Alcaligenes*, *Pseudonocardia*, *Rhodoccus*, *genera*: *Clostridium coccoides*, *Clostridium propionicum*, *Egerthella lenta* in migraine patients compared to healthy controls ↑ Abundance of fungi: *Candida*, *Micromycetes* in migraine patients compared to healthy controls ↑ Load of *Herpes simplex* in migraine patients compared to healthy controls (−) Correlation between *Alcaligenes* and BDI, HAMA, VAS, attacks frequency (−) Correlation between *C. coccoides* and MIDAS, BDI (+) Correlation between *E. lenta* and VAS	Pain severity by VAS Psycho-emotional status by HAMA and BDI Disability by MIDAS Microbiome by chromato-mass spectrometry
[40]	2022	16 migraine + IBS patients	13 IBS patients	Different microbiome structure in migraine + IBS patients compared to IBS patients No statistical difference in microbiome richness and diversity between study and control group ↑ Abundance of 1 genus and ↓ of 5 genera in migraine + IBS patients compared IBS patients No statistical difference in clinical characteristics between study and control group	Microbiome by 16s rRNA gene sequencing Clinical characteristics by MIDAS, PSQI, HAMA, HAMD
[41]	2019	105 migraine with aura female patients	Divided into 2 groups: (i) intestinal dysbiosis (+) (n = 45) (ii) intestinal dysbiosis (−) (n = 60)	↑ Migraine severity, migraine duration, CIMT, TNF-a in intestinal dysbiosis (+) than in intestinal dysbiosis (−)	Microbiome species and intestinal dysbiosis by MALDI-TOF-MS method Migraine severity by MIDAS Subclinical atherosclerosis by CIMT CIMT by ultrasonography

*↑, increase; ↓, decrease; (+), positive; (−), negative; BDI, Beck Depression Inventory; CIMT, carotid intima-media thickness; HAMA, Hamilton Anxiety Scale; HAMD, Hamilton Depression Scale; IBS, irritable bowel syndrome; MALDI-TOF, matrix-assisted laser desorption/ionization-time of flight; MIDAS, Migraine Disability Assessment; MS, mass spectrometry; PSQI, Pittsburgh Sleep Quality Index; Ref., reference; RNA, ribonucleic acid; rRNA, ribosomal RNA; TNF-α, tumor necrosis factor alpha; TOF, time of flight; VAS, visual-analog scale.*

### 3.3 Microbiome and migraine in Mendelian randomization studies

Meng *et al*. [42] used Mendelian randomization analysis to evaluate the relationship between intestinal microbiota and migraine risk. Statistical data were pooled from GWAS, including a total of over 18 thousand participants studied regarding gut microbiota and 37.5 thousand migraine participants. The analysis of the relation between single-nucleotide polymorphisms and both migraine risk and microbiota compounds led to the indication of one family and one genus with a significant role. More precisely, a higher abundance of the genus *Lactobacillus* (*p* = 0.004) and the family *Prevotellacea* (*p* = 0.02) was related to increased and decreased migraine risk, respectively. Similarly, He *et al*. [43] conducted a Mendelian randomization study to find what and how the microbiome may influence migraine risk. The migraine data were obtained from two GWAS databases: the International Headache Genetics Consortium and the Finn-Gen consortium. The inversion variance weighting analysis showed correlations between several microbiota and migraine in general, as well as two major migraine types: migraine with aura and migraine without aura. However, after false discovery rate correction—a higher abundance of only two, family *Bifidobacteriaceae* and order *Bifidobacteriales*, remained to decrease migraine risk, while the rest became insignificant statistically. All studies mentioned in this paragraph, with additional information, were summarized in Table 3 (Ref. [42, 43]).

**Table 3.** A summary of Mendelian randomization studies about the microbiome in migraine.

Ref.	Year	Population	Results	Methods
[42]	2023	18,340 patients (GWAS data for microbiome) 37,500 patients (GWAS data for migraine)	↑ Abundance of family *Prevotellacea* decreased migraine risk	Mendelian randomization from GWAS data
[43]	2023	18,340 patients (GWAS data for microbiome) 4837 migraine with aura and 4833 migraine without aura patients (IHGC GWAS data) 6332 migraine with aura and 8348 migraine without aura patients (FinnGen GWAS data)	↑ Abundance of family *Bifidobacteriaceae* and order *Bifidobacteriales* increased migraine without aura risk according to FinnGen data No significant correlations after correction between microbiome and migraine, migraine with aura, or migraine without aura according to IHGC data and between microbiome and migraine or migraine with aura according to FinnGen data	Mendelian randomization from GWAS data (by IVW) FDR for correction

*↑, increase; FDR, false discovery rate; GWAS, genome-wide association studies; IHGC, International Headache Genetics Consortium; IVW, inverse variant weighting; Ref., reference.*

### 3.4 Microbiome and migraine in preclinical studies

Finally, the last part of the review covers information from preclinical trials. Lanza *et al*. [44] carried out a comprehensive preclinical study exploring the role of short-chain fatty acids (sodium butyrate and sodium propionate) on migraine-related pain, inflammation, and microbiota changes. Nitroglycerine (NTG) was administered to induce migraine models in laboratory mice. Several alterations between the control group and migraine group, including a group treated with topiramate or short-chain fatty acids (supposed to relieve migraine), were revealed. Therefore, it was demonstrated that, apart from alterations in intestinal microbiota between migraine mice and control mice, changes were observed after alleviating migraine with topiramate and presumably with short-chain fatty acids. Tang *et al*. [45] established a migraine model in wild-type mice by NTG administration to study the influence of microbiota changes on migraine pain duration. Mice received antibiotics, probiotics, or sham infusion for 10 days after were treated with NTG. Migraine-like pain was measured by testing orofacial mechanical hyperactivity, and the stimulus was a von Frey filament applied to the forehead (area innervated by the trigeminal nerve). It was demonstrated that mice receiving an antibiotics mixture had significantly prolonged migraine-like pain after NTG injection than migraine mice without antibiotic infusion. Moreover, a further 10-day administration of probiotics may reverse the antibiotic effects on migraine-like pain. Finally, germ-free mice were compared to wild-type mice to confirm the role of microbiota. The migraine pain was substantially prolonged in the former group. Since antibiotics are well known to cause intestinal sterilization, this study showed the crucial role of standard microbiota composition in migraine alleviation. Additionally, antibiotics increased TNF-α upregulation after NTG injection. Therefore, dysbiosis leads to TNF-α increase, which plays a role in triggering migraine pain in the trigeminal nociceptive system.

Another research group used slightly different methods to establish a migraine model. Miao *et al*. [46] conducted a preclinical study to assess the correlation between intestinal microbiota and migraine. A migraine model was established in rats by infusion of an inflammatory soup containing bradykinin, histamine, serotonin, and prostaglandin E2 and compared to the negative control. Both the study and control groups were divided into two subgroups: (i) treated with topiramate and (ii) treated with an equal amount of double-distilled water. Alterations in gut microbiota were shown in migraine model rats compared to negative controls. Moreover, the topiramate administration ameliorated the observed effects, while in the control group, topiramate infusion had no impact on intestinal microbiota. *Lactobacillaceae* (decreased in migraine rats) and *Peptostreptococcaceae* (increased in migraine rats) were the two families that differed significantly. Finally, five genera (*Lactobacillus*, *Colidextribacter*, *Lachnoclostridium*, *Monoglobus*, and *Anaerovorax*) were considered as possible biomarkers due to their different abundance. Among them, *Lactobacillus* was found to be the most prominent after LDA and mean decrease accuracy (MDA) analyses. All studies mentioned in this paragraph, with additional information, were summarized in Table 4 (Ref. [44, 45, 46]).

**Table 4.** A summary of preclinical research on microbiome in migraine.

Ref.	Year	Population	Comparison	Results	Methods
[44]	2022	Sodium propionate or sodium butyrate-treated migraine model mice (in different doses)	Negative control mice Migraine model mice Sumatriptan-treated migraine model mice	↑ Latency time in sodium propionate or sodium butyrate treated mice in comparison to migraine model mice ↑ Abundance of phylum *Firmicutes* and ↓ of phylum *Bacteroidetes* in migraine mice (regardless sodium propionate or sodium butyrate receiving) than in negative control mice ↑ Abundance of genus *Alistipes* in non-treated migraine mice to sodium propionate, sodium butyrate or sumatriptan-treated ↑ Abundance of genera *Chitinophagaceae. Burkholderiales* and species *Prevotella* in negative control mice compared to others	Hargreaves Test for thermal nociceptive thresholds Von Frey Test for mechanical allodynia microbiome by 16s rRNA sequencing
[45]	2020	Antibiotics-receiving migraine model mice (wild-type and germ-free)	Negative control migraine model mice Probiotics-receiving migraine model mice (wild-type and grem-free)	↑ Head withdrawal threshold in antibiotics-receiving migraine model mice after nitroglicerine injection Reversion of antibiotics’ effects on head withdrawal threshold after probiotics infusion (↑ effects in germ-free than wild-type mice) No statistical difference between negative control, antibiotics—and probiotics—receiving migraine model mice in head withdrawal threshold before nitroglicerine injection	Migraine-like pain by head withdrawal threshold
[46]	2022	Migraine model rats treated (n = 8) or untreated (n = 8) with topiramate	Negative control rats treated (n = 8) or untreated (n = 8) with topiramate	Altered microbiome in migraine model rats (ameliorated by topiramate) *Lactobacillus* found as the most prominent biomarker	Migraine model by infusions of inflammatory soup Cephalic allodynia and topiramate effect by periorbital mechanical threshold (Von Frey Test) and nociception-related behaviors Microbiome by 16s rRNA gene sequencing

*↑, increase; ↓, decrease; n, number; Ref., reference; RNA, ribonucleic acid; rRNA, ribosomal RNA.*

### 3.5 Additional findings after literature review: probiotics in migraine prophylaxis

The selection process of the review led us to find some research that did not raise the issue of microbiome differences directly, but provided interesting observations about the influence of probiotics supplementation on migraine courses. After following the PRISMA guidelines, we found only two randomized controlled trials that matched all inclusion criteria. Both studies included 159 patients, 83 in the probiotics groups and 76 in the placebo groups. Patients’ ages range from 18 to 70 years old. All included participants suffered from migraine, either chronic or episodic.

Three different parameters were taken into consideration in both studies: (i) migraine severity, (ii) migraine frequency per month, and (iii) attack duration. Importantly, although there are only two trials in this analysis, different results were obtained, including either statistically significant or insignificant results for the same parameter. Therefore, a statistical analysis of the results was performed to answer those alterations. For the two former features, migraine severity and frequency, the heterogeneity test showed high heterogeneity (*p* < 0.001, Q = 26.16, *I*^2^ = 92.36%, τ^2^ = 1.19 for severity and *p* = 0.003, Q = 11.65, *I*^2^ = 82.84%, τ^2^ = 0.47 for frequency), and therefore, a random effect model was applied. The latter characteristic presented low heterogeneity between all results (*p* = 0.426, Q = 1.71, *I*^2^ = 0.00%, τ^2^ = 0.00); thus, a fixed effect model was chosen here.

#### 3.5.1 Probiotics’ influence on migraine severity

Different conclusions were drawn by the two research groups. Ghavami *et al*. [47] compared 40 migraine patients receiving probiotic supplementation for 12 weeks to 40 individuals from the control group receiving a placebo for the same time. Moreover, a comparison was performed before and after the observation time. Migraine severity appeared not to differ significantly after probiotics therapy (*p* = 0.497), and unsurprisingly, the same observations were made for the control group (0.331). The changes between the baseline and the end of the trial were not significantly different between probiotics and placebo groups (*p* = 0.168).

On the other hand, Martami *et al*. [48] divided their study into two parts: (i) chronic migraine patients treated with probiotics (n, number = 21) or placebo (n = 18) and (ii) episodic migraine patients treated equally (n = 40; 22 in probiotics group, and 18 in placebo group). Differently to the previous research, in both groups, migraine severity appeared to lower after 8 weeks of probiotics intake (*p* < 0.001 each), while no statistical change observed in the placebo groups (*p* = 0.298 for chronic migraine and *p* = 0.579 for episodic migraine). Consistently, differences between probiotic and placebo groups were analyzed in a one-way analysis of variance (ANOVA) test and were statistically significant (*p* < 0.001).

Finally, differences between probiotics and placebo groups in changes observed after several weeks of observation were assessed overall. It appeared, after pooling, that the study and control groups did not differ significantly (*p* = 0.069), even though, in the second research, the difference was statistically significant for both episodic and chronic migraine patients. However, the calculated *p*-value is close to the border of significance for the analysis of migraine severity, differing between probiotics and placebo receivers. Hedges’ *g* effect size for this analysis was 1.10 (standard error (SE) = 0.61), where 0.00 represents no change. The forest plot is shown below (Fig. 3, Ref. [47, 48]). Therefore, more high-quality trials are needed in this specific field.

**Fig. 3.** Forest plot of one of the efficacy outcomes. Differences between probiotics and placebo receivers in the change in migraine severity before and after intervention, with mean difference and confidence intervals. Pooled effect size = 1.10 (standard error = 0.61). The point 0.00 represents no change. CM, chronic migraine; EM, episodic migraine.

#### 3.5.2 Probiotics’ influence on migraine frequency

The second issue raised is the change in migraine frequency after the course of probiotics. In the trial conducted by Ghavami *et al*. [47], migraine frequency decreased in the study group (*p* < 0.001), while migraine sufferers receiving a placebo did not experience any significant difference (*p* = 0.331). Contrary to the migraine severity, the change between the baseline and the end of the trial differed significantly between the study and control groups, and was more significant in the former (*p* = 0.011). In the second analyzed study [48], this time consistently with the first one, probiotics decreased migraine frequency in chronic and episodic migraine patients (*p* < 0.001 each). The results of the placebo group did not reach statistical significance (*p* = 0.449 in chronic migraine and *p* = 0.749 in episodic migraine). Thus, unsurprisingly, the difference between the study and control groups in the change achieved after intervention was statistically significant (*p* < 0.001). Hedges’ *g* effect size for this analysis was 1.22 (SE = 0.41), where 0.00 represents no change.

The calculations after pooling results confirmed the observations made by both research groups (shown graphically on the forest plot—Fig. 4 (Ref. [47, 48])). The change in migraine frequency was significantly greater in patients treated with probiotics than those receiving placebo (*p* = 0.003).

**Fig. 4.** Forest plot of one of the efficacy outcomes. Differences between probiotics and placebo receivers in the change in migraine frequency before and after intervention, with mean difference and confidence intervals. Pooled effect size = 1.22 (standard error = 0.41). The point 0.00 represents no change. CM, chronic migraine; EM, episodic migraine.

#### 3.5.3 Probiotics’ influence on attacks’ duration

The last analyzed characteristic was the duration of migraine attacks. Ghavami *et al*. [47] did not observe significant differences before and after probiotics or placebo intake in migraine patients (*p* = 0.131, *p* = 0.094, respectively), nor in the observed change between those two groups (*p* = 0.327). In the study conducted by Martami *et al*. [48], the results appeared to differ in probiotics recipients depending on the migraine type. More precisely, the difference before and after 8 weeks of observation was significant for chronic migraine (*p* = 0.034) but not episodic migraine (*p* = 0.370). On the other hand, no significant change in the placebo group was observed regardless of migraine type (*p* = 0.109 for chronic migraine and *p* = 0.397 for episodic migraine). Finally, the study and control groups did not differ significantly in terms of the observed change (*p* = 0.149). Hedges’ *g* effect size for this analysis was 0.18 (SE = 0.15), where 0.00 represents no change.

After pooling data from the analyzed studies, the no difference was observed between probiotics and placebo groups in terms of decrease in the duration of attacks before and after intervention (*p* = 0.248). The forest plot is shown below (Fig. 5, Ref. [47, 48]).

**Fig. 5.** Forest plot of one of the efficacy outcomes. Differences between probiotics and placebo receivers in the change in attacks’ frequency before and after intervention, with mean difference and confidence intervals. Pooled effect size = 0.18 (standard error = 0.15). The point 0.00 represents no change. CM, chronic migraine; EM, episodic migraine.

## 4. Conclusions

Microbiota may differ in migraine sufferers at the level of families, species, and genera. In some articles, microbiota, in general, have been studied, whereas others have described some particular ones. Overall, the microbiome has been significantly altered in migraine. The alterations may be both a cause and a result of the disease, that’s to say, being one of the factors contributing to the disease development, as well as an effect of the changes in the human organism triggered by a disorder. Therefore, these findings are essential for causative as well as symptomatic treatment. Moreover, microbiota alterations influenced migraine clinical presentation, influencing characteristics such as migraine severity, migraine frequency, psycho-emotional status, and impact on patient’s quality of life (Fig. 6). Some genera can be correlated with the alleviation or aggravation of migraine course. Therefore, attempts to modify intestinal microbiota in migraine patients may be considered a novel therapy and require clinical trials to verify this hypothesis.

**Fig. 6.** A graphical summary of the aspects influenced by microbiota alterations in migraine patients. MIDAS, migraine disability scale (a scale assessing headache-related disability over the last three months).

These observations are confirmed by Mendelian-randomization studies, which revealed that some genera are directly linked to migraine, increasing or decreasing the risk of its development. Therefore, among the factors with a significant role in the correlation between migraine and microbiome are not only environmental ones, as the intestinal microbiome is known to be influenced by external factors, but also genetic ones.

Migraine and microbiota analyses were also a topic of some preclinical trials; however, their number is very limited. Unsurprisingly, microbiota has been altered in laboratory animals with established migraine models. Nevertheless, an interesting observation is that specific migraine treatment appears to reverse changes in the microbiome induced by migraine; for instance, in two preclinical studies, topiramate not only alleviated migraine but also undid the changes in gut microbiota. This supports the theory of direct correlation between migraine and gut microbiota alterations, regardless of some unspecified indirect factors.

Undoubtedly, the microbiota-gut-brain axis, described in detail in the previous parts of the article, plays a crucial role. The undeniable connection between the digestive tract and the neurological system may explain the abovementioned correlations. Interfering through three different pathways: endocrine, immune, and neurological, gut microbiota sends diverse signals to the brain, in some cases, probably with harmful effects. However, more precise mechanisms are not thoroughly described and are a topic for further investigation.

Analyzing the role of the microbiome in migraine raises the question of whether probiotics can be used in migraine treatment or prevention. Some observations suggested that supplementing probiotics can alleviate migraines by modifying the intestinal microbiota. However, the data appeared to be inconsistent between the studies. Randomized controlled trials were analyzed to summarize the available data. Probiotics use appeared to decrease migraine severity; therefore, their use can be beneficial as an addition to standard migraine medications and prevention habits. No significant changes after probiotics use were observed regarding migraine frequency and duration of attacks. However, the results were close to the statistical significance for the analysis of probiotics’ role in decreasing migraine frequency compared to placebo treatment. The results remain slightly unclear, even after statistical analysis. Our findings align with prior reviews that highlighted the potential role of gut microbiota in migraine pathophysiology [27, 49]. However, these and other reviews did not provide a quantitative synthesis of interventional trials. Our meta-analysis contributes novel pooled estimates of the effect of probiotics on migraine outcomes, addressing this gap; however, with a very limited number of studies. Undoubtedly, more randomized controlled trials are needed to assess whether probiotics supplementation should be recommended in migraine patients.

Although this work is comprehensive, it leaves some uncertainties and shows directions for further research. Nevertheless, the role of the microbiome in migraine, although not fully understood, is undeniable. Future studies should focus on large, multicenter randomized controlled trials evaluating probiotic interventions with well-defined clinical endpoints. Incorporating microbiome sequencing and metabolomic profiling could help explain underlying mechanisms and, thus, identify potential microbial biomarkers associated with migraine improvement. Such an approach would enable more targeted, personalized therapeutic strategies in this field.

## 5. Limitations

This work does not remain without limitations. The main limitation regarding the included studies is their limited number, especially the number of randomized controlled trials for meta-analysis is very low. Additionally, the included studies had small samples of patients, which could cause bias in the obtained results.

Regarding the methodology of the work, there are also some limitations. Two databases were screened, with the risk that some studies, not included in any of them, could have been omitted. Also, the main searches were conducted in 2024 and following the protocol registered in PROSPERO, the decision was not to expand them; however, we performed additional searches in April, May, and June 2025 to minimize the risk of significant bias. Furthermore, including studies only in the English language may lead exclusion of important studies written in other languages. The statistical analyses made using Microsoft Excel instead of dedicated meta-analytical software should be considered a limitation. However, given the relatively small number of included studies and outcomes, we believe that our analytical approach was sufficient for the scope of this review. Additionally, the potential for reporting and publication bias can not be fully excluded, given the small number of included studies and the positive outcome focus typical for early-phase trials in this domain. While no formal tests were conducted due to limited power (n < 10), this limitation should be considered when interpreting the pooled results.

Despite all the above, we believe that the provided work brings an important value to the field of migraine management and brings us closer to a better understanding of migraine pathogenesis.

## Supplementary Material

Supplementary material associated with this article can be found, in the online version, at https://files.jofph.com/ files/article/1966335432042921984/attachment/ Supplementary%20material.docx.


