# Supplementary material for: Gut microbiota, probiotics, and migraine: a clinical review and meta-analysis
Source: J Oral Facial Pain Headache. 2025 Sep 12;39(3):13–26. doi: 10.22514/jofph.2025.043 (PMC12520441; doi:10.22514/jofph.2025.043)
Supplement: Supplementary file 1 [file Supplementary-material.docx]

Supplementary material

Supplementary Table 1. A brief presentation of the full search query, search dates, and language limits in each of the article parts.

| Methodology Part | Migraine & Microbiome | Migraine & Probiotics |
| --- | --- | --- |
| Full search query | (“migrain”[All Fields] OR “migraine disorders”[MeSH Terms] OR (“migraine”[All Fields] AND “disorders”[All Fields]) OR “migraine disorders”[All Fields] OR “migraine”[All Fields] OR “migraines”[All Fields] OR “migraine s”[All Fields] OR “migraineous”[All Fields] OR “migrainers”[All Fields] OR “migrainous”[All Fields]) AND (“gastrointestinal microbiome”[MeSH Terms] OR (“gastrointestinal”[All Fields] AND “microbiome”[All Fields]) OR “gastrointestinal microbiome”[All Fields] OR (“intestinal”[All Fields] AND “microbiota”[All Fields]) OR “intestinal microbiota”[All Fields] OR (“gastrointestinal microbiome”[MeSH Terms] OR (“gastrointestinal”[All Fields] AND “microbiome”[All Fields]) OR “gastrointestinal microbiome”[All Fields] OR (“gut”[All Fields] AND “microbiota”[All Fields]) OR “gut microbiota”[All Fields]) OR (“gastrointestinal microbiome”[MeSH Terms] OR (“gastrointestinal”[All Fields] AND “microbiome”[All Fields]) OR “gastrointestinal microbiome”[All Fields] OR (“intestinal”[All Fields] AND “microbiome”[All Fields]) OR “intestinal microbiome”[All Fields]) OR (“gastrointestinal microbiome”[MeSH Terms] OR (“gastrointestinal”[All Fields] AND “microbiome”[All Fields]) OR “gastrointestinal microbiome”[All Fields] OR (“gut”[All Fields] AND “microbiome”[All Fields]) OR “gut microbiome”[All Fields])) | (“migrain”[All Fields] OR “migraine disorders”[MeSH Terms] OR (“migraine”[All Fields] AND “disorders”[All Fields]) OR “migraine disorders”[All Fields] OR “migraine”[All Fields] OR “migraines”[All Fields] OR “migraine s”[All Fields] OR “migraineous”[All Fields] OR “migrainers”[All Fields] OR “migrainous”[All Fields]) AND (“probiotic s”[All Fields] OR “probiotical”[All Fields] OR “probiotics”[MeSH Terms] OR “probiotics”[All Fields] OR “probiotic”[All Fields]) |
| Search dates | 13 January 2024–16 April 2024 | 18 February 2024 |
| Additional searches dates | 01 April 2025–05 June 2025 | |
| Language limits | Only English language articles | Only English language articles |

Supplementary Table 2. GRADE summary of findings with the assessment of the certainty of evidence.

| Outcome | No. of Participants (Studies) | Effect (95% CI) | Certainty of Evidence | Rationale/Comments |
| --- | --- | --- | --- | --- |
| Migraine frequency (attacks/month) | 106 (2 RCTs) | ↓ ~1.3 attacks/month (CI varies across studies) | Low | Both studies showed a reduction in frequency. Limitations include small sample sizes, some risk of bias, and high heterogeneity. |
| Migraine severity (VAS score) | 106 (2 RCTs) | ↓−0.83 (95% CI: −1.45 to −0.20) | Low | Statistically significant effect in pooled analysis. Downgraded for imprecision and some methodological limitations. |
| Inflammatory biomarkers (CRP, TNF-α, IL-6) | 106 (2 RCTs) | ↓ CRP, TNF-α, IL-6 (varied results) | Very Low | Results inconsistent and short-term. Biomarker assessment not standardized; unclear clinical significance; indirect evidence. |

CI: confidence interval; RCT: randomized controlled trials; VAS: visual analog scale; CRP: C-reactive protein; TNF-α: tumor necrosis factor alpha; IL-6: interleukin 6.
